# PAIN AND MOBILITY OF OLDER ADULTS IN MEDICAL AND SURGICAL UNITS: AN EHR-BASED DATA ANALYSIS

**DOI:** 10.1093/geroni/igae098.4039

**Published:** 2024-12-31

**Authors:** Urszula Snigurska, Sarah Ser, Mattia Prosperi, Ragnhildur Bjarnadottir, Robert Lucero, Todd Manini

**Affiliations:** University of Florida Clinical and Translational Science Institute, Gainesville, Florida, United States; University of Florida College of Public Health and Health Professions & College of Medicine, Gainesville, Florida, United States; College of Public Health and Health Professions & College of Medicine, Gainesville, Florida, United States; University of Florida College of Nursing, Gainesville, Florida, United States; University of California Los Angeles, Los Angeles, California, United States; University of Florida College of Medicine, Gainesville, Florida, United States

## Abstract

Pain accelerates age-related decline in mobility. One in five hospitalized older adults have pain. Pain experience varies by the type of hospitalization. The purpose of this study was to describe pain and mobility in hospitalized older adults. We hypothesized that pain would be higher in patients with lower mobility. We analyzed electronic health record data from an academic health center in Florida between 2012 and 2021. We used the mean pain score and mean score on the mobility subscale of the Braden Scale for each encounter. We used the Wilcoxon Rank-Sum Test and Pearson’s correlation coefficient to measure the differences and relationship between pain and mobility. The cohort included 19,854 older adults (13,878 medical and 5,976 surgical patients). The mean mobility score was 3.05 ± 0.57 in medical and 2.88 ± 0.51 in surgical patients (W = 48697407, p < 0.001). The mean pain score was 1.93 ± 2.72 in medical and 2.25 ± 2.61 in surgical patients (W = 36893487, p < 0.001). There was no correlation between pain and mobility in either medical (r = 0.01, p = 0.427) or surgical patients (r = 0.02, p = 0.085). Mean pain intensity may not be associated with mobility in hospitalized older adults. Instead, the highest level of pain or uncontrolled pain may be associated with mobility decline. Our findings provide motivation for a future study to account for confounders of the relationship between pain and mobility, such as analgesics, and temporality of pain and mobility assessments in the hospital.

